# *Notes from the Field:* Enhanced Surveillance for Raccoon Rabies Virus Variant and Vaccination of Wildlife for Management — Omaha, Nebraska, October 2023–July 2024

**DOI:** 10.15585/mmwr.mm7341a4

**Published:** 2024-10-17

**Authors:** Sydney R. Stein, Andrew J. Beron, Kathleen M. Nelson, Emma Price, Sergio E. Rodriguez, Victoria Shelus, Ann Carpenter, Amy R. Hess, Cassandra Boutelle, Clint N. Morgan, Crystal M. Gigante, Christina L. Hutson, John D. Loy, Duan S. Loy, Chad Wetzel, Justin Frederick, Lindsay Huse, Lillian Orciari, Richard B. Chipman, Ryan M. Wallace, Matthew Donahue, Bryan F. Buss

**Affiliations:** ^1^Epidemic Intelligence Service, CDC; ^2^Nebraska Department of Health and Human Services; ^3^Division of High-Consequence Pathogens and Pathology, National Center for Emerging and Zoonotic Infectious Diseases, CDC; ^4^Wildlife Services, Animal and Plant Health Inspection Service, U.S. Department of Agriculture; ^5^Laboratory Leadership Service, CDC; ^6^University of Nebraska-Lincoln, Veterinary Diagnostic Center, Lincoln, Nebraska; ^7^Douglas County Health Department, Omaha, Nebraska; ^8^Career Epidemiology Field Officer Program, CDC.

**Summary**

**What is already known about this topic?**

Movement of wildlife has facilitated the spread of non-bat rabies virus variants into new geographic areas of the United States, with major implications for human health.

**What is added by this report?**

A stray kitten died of raccoon rabies virus variant 850 miles west of this variant’s known range. Rabies virus variant typing led to prompt surveillance and mitigation efforts, which prevented a potential rabies outbreak in the Midwestern United States.

**What are the implications for public health practice?**

Routine vaccination of domestic animals and rabies virus variant typing are crucial to preventing, identifying, and mitigating future translocation events.

On September 28, 2023, a kitten aged approximately 6 weeks found in Omaha, Nebraska, had test results positive for rabies at the Nebraska Veterinary Diagnostic Center (NVDC) after dying with neurologic signs and having bitten and scratched its caretakers. Preliminary investigation identified 10 exposed persons for whom postexposure prophylaxis (PEP)^†^ was recommended. Subsequent variant-typing by NVDC yielded a presumptive positive result for the Eastern raccoon rabies virus variant (RRVV), which CDC confirmed on October 6.

Rabies is a fatal, yet preventable, viral disease primarily transmitted through the bite of infected animals; after exposure, the incubation period can last from weeks to months. Globally, dogs are associated with 95% of the annual estimated 70,000 human deaths from rabies. However, in the United States, because of robust rabies control efforts that eliminated canine rabies virus variant and the widespread availability of PEP, fewer than 10 persons die from rabies each year. Rabies is enzootic in bats across the continental United States as well as in geographically distinct non-bat mammal reservoirs, including raccoons, skunks, and foxes (*1*). Initially found only in the southeastern United States, RRVV was introduced into mid-Atlantic states in 1977, after the translocation of wild raccoons from Florida (*2*), resulting in one of North America’s most important documented wildlife epizootics^§^ (*3*,*4*). As RRVV spread along the east coast, the number of human rabies exposures rose substantially, resulting in increased demand for PEP. Because this kitten of unknown origin was found approximately 850 miles (1,368 km) west of the known range of RRVV, and transmission to local wildlife in Nebraska could not be excluded, a coordinated multiagency response was initiated to determine if local transmission of RRVV was occurring and to implement a wildlife vaccination program.

## Investigation and Outcomes

Beginning October 14, 2023, the Nebraska Department of Health and Human Services, Douglas County Health Department, CDC, and U.S. Department of Agriculture (USDA) initiated field activities. Steps included simultaneously attempting to identify the kitten’s origin, identifying additional exposures to the kitten, implementing an enhanced rabies surveillance program, and mitigating further spread through wildlife vaccination. Investigators interviewed community members, examined social media platforms for reports of animals exhibiting behavior suggestive of neurologic disease,^¶^ and disseminated public messaging through neighborhood flyers, social media accounts, and press conferences. Despite the expansive outreach, no further exposures or information regarding the origin of the kitten were identified.

Investigators established a field laboratory, with a focus on testing animals identified within a 6.2-mile (10-km) radius of the index RRVV case that were found dead or were exhibiting behavior suggestive of neurologic disease. A commercially available point-of-care test** was used to conduct rapid field testing for rabies. Brainstem specimens that yielded a presumptive positive test result and a subset of negative samples underwent confirmatory testing^††^ at CDC. Among specimens tested from 515 animals through July 31, 2024, none were positive for rabies virus, including those from 350 (68.0%) raccoons, 46 (8.9%) skunks, 63 (12.2%) feral cats, and 56 (10.9%) animals of other species. This activity was reviewed by CDC, deemed not research, and was conducted consistent with applicable federal law and CDC policy.^§§^

During October 23–November 2, USDA initiated a trap-vaccinate-release campaign^¶¶^ intended for raccoons and striped skunks across a 37-mi^2^ (96-km^2^) area, followed by a more intense placement of oral rabies vaccine (ORV) baits*** across a 65-mi^2^ (162-km^2^) area of Omaha during November 1–4. In total, 757 raccoons, 42 skunks, four feral cats, and one red fox were vaccinated through the trap-vaccinate-release campaign, and 18,000 ORV vaccine baits were placed. 

## Preliminary Conclusions and Actions

This case represents the westernmost detection of RRVV in a stray or wild animal in the United States. In 2017, an RRVV-infected cat was detected in Ohio (*5*); however, that cat was owned and documented to come from an enzootic area and had no potential contact with wildlife or stray animals. Unlike the response to the 2017 detection, this response required robust rabies surveillance and implementation of a multifaceted wildlife vaccination campaign to address potential introduction of RRVV into local wildlife populations. No further detection of RRVV over a 10-month surveillance period, accompanied by concurrent intensive and focal wildlife vaccination efforts, suggests lack of establishment of RRVV in wildlife in Nebraska. Timely variant typing of the rabies virus infecting this kitten prompted a large-scale response to prevent RRVV establishment in Nebraska wildlife. Maintaining high levels of rabies vaccination for domestic animals and increasing variant typing for rabies virus–positive specimens, particularly those from domestic animals and non-reservoir wildlife species, are crucial to preventing, identifying, and mitigating future translocation events.

## Footnotes

† For persons who have never been vaccinated against rabies, PEP comprises a combination of human rabies immune globulin and a first rabies vaccine dose administered following a potential or confirmed rabies exposure; subsequent rabies vaccine doses are administered on days 3, 7, and 14. For immunocompromised persons, an additional vaccine dose is given on day 28. For persons previously vaccinated against rabies, PEP consists of rabies vaccination on days 0 and 3. https://www.cdc.gov/rabies/hcp/prevention-recommendations/post-exposure-prophylaxis.html

§ In New York, an approximately 100-fold increase in annual rabies PEP administrations occurred within 5 years of RRVV detection. To aid in preventing the westward expansion of RRVV, Congress has appropriated approximately $500 million to USDA for implementation of wildlife rabies management strategies.

¶ Behavior in wildlife suggestive of neurologic disease, which might be indicative of rabies virus infection, includes but is not limited to hypersalivation, paralysis, lethargy, abnormal aggression, abnormal vocalizations, and diurnal activity of nocturnal species.

** Antigen Rapid Rabies Ag Test Kit (BioNote).

†† The BioNote lateral flow assay (LFA) was chosen as a screening test for this response because of its suitability for use in the field for rabies testing. Because LFA is not yet a recognized rabies diagnostic test in the United States, all samples with a presumptive positive result and ≥20% of randomly selected samples with negative test results underwent confirmatory testing by direct fluorescent antibody or LN34 pan-lyssavirus real-time polymerase chain reaction testing by CDC.

§§ 45 C.F.R. part 46.102(l)(2), 21 C.F.R. part 56; 42 U.S.C. Sect. 241(d); 5 U.S.C. Sect. 552a; 44 U.S.C. Sect. 3501 et seq.

¶¶ Healthy animals within an approximately 3-mi (5-km) radius of the index case received a 1-mL intramuscular dose of IMRAB 3 inactivated rabies vaccine (Boehringer Ingelheim Animal Health USA Inc.).

*** RABORAL V-RG (Boehringer Ingelheim Animal Health USA Inc.) baits were distributed within an approximately 6-mi (10-km) radius of the index case location, stopping at the eastern border with Iowa and the Missouri River.
